# Prediction of air temperature and humidity in greenhouses via artificial neural network

**DOI:** 10.1371/journal.pone.0325650

**Published:** 2025-06-09

**Authors:** Caixia Yan, Ta Na, Qi Zhen, Yunfeng Sun, Kunyu Liu

**Affiliations:** 1 College of Mechanical and Electrical Engineering, Inner Mongolia Agricultural University, Hohhot, China; 2 College of Energy and Transportation Engineering, Inner Mongolia Agricultural University, Hohhot, China; Agricultural Sciences and Natural Resources University of Khuzestan, IRAN, ISLAMIC REPUBLIC OF

## Abstract

Accurate prediction of greenhouse temperature and relative humidity is critical for developing environmental control systems. Effective regulation strategies can help improve crop yields while reducing energy consumption. In this study, Multilayer Perceptron (MLP) and Radial Basis Function (RBF) networks were used for short-term prediction of temperature and relative humidity in a double-film greenhouse. The prediction models used indoor soil temperature, light intensity, and historical measurements of temperature and humidity from the previous 10 minutes as inputs. Results show that the MLP model with Levenberg-Marquardt optimization performs best in predicting the current temperature and humidity, with an RMSE of 0.439°C and R^2^ of 0.997 for temperature prediction and an RMSE of 1.141% and R^2^ of 0.996 for relative humidity prediction. For 30-minute short-term prediction, the Bayesian optimized RBF model showed better temperature prediction with an RMSE of 1.579°C and an R^2^ of 0.958, while the MLP model performed better in relative humidity prediction with an RMSE of 4.299% and an R^2^ of 0.948. This study provides theoretical support for advancing the intelligent regulation of greenhouse environmental factors in cold and arid regions, and the application of predictive models to intelligent environmental management systems could help optimize cultivation practices and energy efficiency.

## 1. Introduction

Greenhouses are widely used systems for artificially creating a suitable growth microclimate environment for plants. Greenhouse environmental factors such as temperature and humidity are critical environmental factors influencing plant development, quality, and production quantity [1]. More specifically, the exposure of plants to extreme temperatures can cause heat damage or frost damage, and an increase in relative humidity may exacerbate fungal diseases and affect the uptake and utilization of calcium and the normal water balance of plants [2]. Therefore, these factors need to be considered when growing and managing plants, and appropriate measures need to be taken to protect plants from the adverse effects of high-humidity environments. Greenhouse climatic control necessitates the consideration of complex, nonlinear and strong coupling systems, where variables are highly dependent on external climate conditions as well as structure type, design and orientation [3].

**Fig 1:**
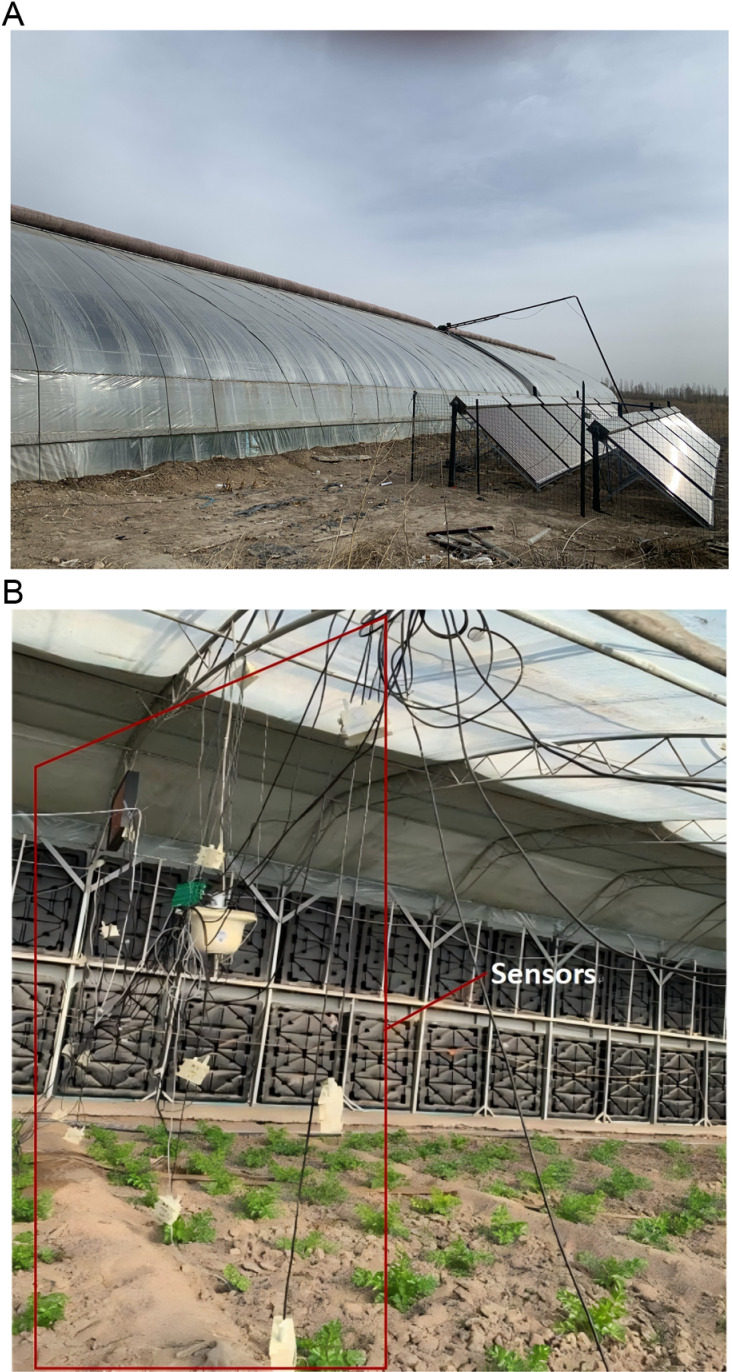
Pictures of the test greenhouse.(a) Appearance of the test greenhouse. (b) Interior of the test greenhouse.

The Hohhot region, where the experimental study was conducted, is a typical cold and arid region in China, where the average temperature in January in winter is about −15°C, and the extreme low temperature exceeds −30°C. The region is vigorously developing facility agriculture and promoting the “Vegetable Basket Project”. In 2024, newly constructed facilities covered 690 ha (10,350 mu), while vegetable cultivation spanned 3,600 ha (54,000 mu).solar greenhouses constitute a vital component of winter vegetable provisioning systems, and some greenhouses are planted with high-value cultivars such as medicinal herbs and premium fruits to increase farmers’ income. Presently, the operation of traditional greenhouses (Fig 1) is mainly dependent on manual expertise, with low automation. Consequently, precise regulation of greenhouse temperature and relative humidity is imperative to ensure optimal growing environments for plants, enhance crop yields, and mitigate losses caused by climatic fluctuations. Obviously, building a precise model of the inside greenhouse climate is an important way to achieve efficient microclimate management [4,5].

Modeling of solar greenhouses can be based on either physical laws or system identification methods. Modeling via system identification methods is more advantageous than physical modeling and is more responsive to the requirements of environmental factor regulation [6,7]. The rapid development of Artificial Intelligence has enabled it to play an important role in a variety of application scenarios [8–10]. As a typical data-driven tool, Artificial Neural Networks (ANN) are widely used to predict environmental factors such as temperature and humidity in greenhouses due to their excellence in capturing nonlinear relationships between multivariate inputs such as light, humidity and temperature [11,12]. Castañeda and Castaño et al. [13] developed a Levenberg-Marquardt optimized multilayer perceptron (MLP) model, achieving remarkable determination coefficients of 0.9549 (winter) and 0.9590 (summer) for indoor temperature prediction. This implementation effectively addressed frost control challenges, validating ANN’s practical utility in complex agricultural environments. Petrakis et al. [14] used a BP neural network to predict temperature and relative humidity in a greenhouse, and the inputs were 10 variables, including historical indoor temperature and relative humidity. The number of nodes in the hidden layer of the BP neural network was derived through extensive experimental comparison. Zhang et al. [15] used the sparrow search algorithm (SSA) to optimize the radial basis function (RBF) network to carry out a simulation study on the temperature and humidity of greenhouses. The coefficient of determination (R^2^) of the temperature and humidity was approximately 0.86, but the algorithm has a relatively large number of input variables. Hayoung et al. [16] predicted the indoor temperature and relative humidity of an eight-span greenhouse via a MLP model. The effects of different numbers of hidden layers and nodes on the prediction accuracy were discussed, and the optimal network structure with four hidden layers and 128 nodes for air temperature (R^2^ = 0.988) and with four hidden layers and 64 nodes for relative humidity (R^2^ = 0.990) was determined. However, too many hidden layer nodes can lead to an overly large network structure, making the computation slower or less pervasive [17]. Mohmed et al. [18] proposed LM-MLP model to predict the maximum and minimum temperature during two different seasons (warm and cold), the mean absolute error between the predicted and the measured temperatures was 0.6833°C. Liu et al. [19] established a BP neural network model to predict the temperature variation trend in greenhouses for the next 1–7 days on the basis of daily meteorological data from Tianjin from 2011–2013 in the winter. Yue et al. [20] proposed a model to predict the temperature and humidity of a greenhouse based on improved LM-RBF model. The model uses the inside and outside meteorological data of the greenhouse as inputs and the temperature and humidity in a greenhouse as outputs, but they do not consider the effects of historical indoor temperature and humidity on the prediction accuracy of the system. Yu et al. [21] established a new prediction model based on a least squares support vector machine (LSSVM) using the environmental factors of Shandong Province to predict the temperature of a greenhouse. These authors considered the historical inside temperature and predicted temperature well but did not predict indoor humidity. Zou et al. [22] took the indoor temperature and relative humidity of the previous moment and the external meteorological data as the input vector (6 in total) of the prediction model and established a prediction model of temperature and humidity in a greenhouse.

Despite recent advancements in greenhouse microclimate modeling, there has been less research on short-term predictions of temperature and relative humidity for traditional double-layer film greenhouses for cold and arid regions in China. This paper employs two representative artificial neural network models (MLP and RBF) to predict the temperature and relative humidity inside the greenhouse 30 minutes after its initial state. Initially, the input variables were optimized using spearman correlation analysis to eliminate environmental variables with low correlation. Subsequently, a new integration of Local Sensitivity Analysis (LSA) and Kendall’s W coefficient of concordance was performed to verify the statistical significance of the results of the input variables ranked according to their importance. Secondly, the effects of training sample partitioning methods (60%−80%) on the output results of MLP and RBF prediction models were investigated, and the dataset partitioning schemes of the two prediction models were optimized, respectively. Finally, the key hyperparameters and training algorithms of the MLP and RBF are optimized separately, and the final performance of both models is compared to select the optimal model. In this study, the 5-fold cross-validation method was used in all predictive models to ensure the stability of the results. The findings of this study have the potential to provide technical support for high-precision environmental regulation and to promote the transformation and upgrading of traditional facility agriculture to smart agriculture.

## 2. Materials and methods

### 2.1 Greenhouse and measurements

All the experiments were based on a greenhouse (experimental area: 25 m × 8 m, 200 m^2^) located in Hohhot, Inner Mongolia, northwestern China, Hailiu Village Science and Technology Park of Inner Mongolia Agricultural University (lat. 40.68_N, long. 111.38_E). The crop grown in the greenhouse during the experiment was celery. The greenhouse is equipped with inner and outer double-layer film and inner and outer thermal insulation quilts. The back wall is a brick–concrete structure and has an external thermal insulation layer that is 8 cm thick. In addition, a solar heating system was added to the greenhouse. The exterior and interior of the greenhouse are shown in Fig 1(a) and Fig 1(b). Compared with other types of greenhouses, the experimental greenhouse’s minimum temperature is above 0°C, which is suitable for the cold, dry areas of northern China. When the sunshine is sufficient at approximately 9:00 in the morning in winter, the outer quilt and the inner quilt are opened, so that the solar greenhouse can absorb more solar radiation, which can promote plant growth and make the solar greenhouse temperature rise rapidly, inner film is opened at approximately 10:30 a.m. according to weather conditions and the top vent is opened at noon. At approximately 3:30 p.m., the solar radiation gradually weakened, and the inner and outer quilts and inner film were put down, which slowed the heat loss of the solar greenhouse to the outside and played a role in heat preservation. The solar heating circulation system starts working at approximately 8:00 p.m.

**Fig 2:**
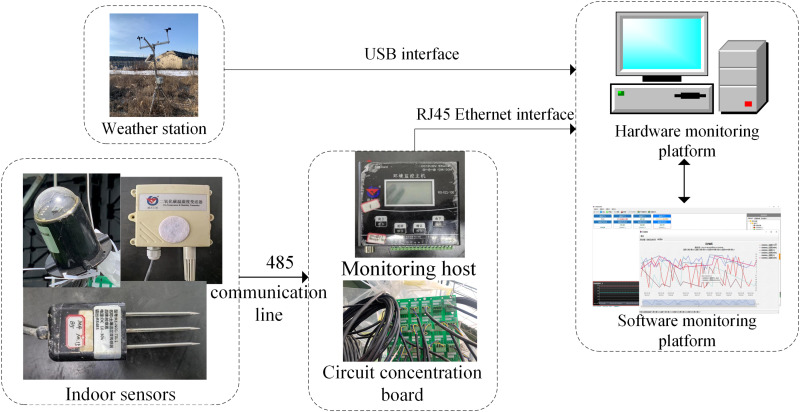
Greenhouse environmental factor collection system.

Indoor environmental factors were measured using appropriate sensors: air temperature (−10°C to 50°C, ± 0.5°C), relative humidity (0–95% RH, ± 3%) and CO_2_ concentration (0–5000 ppm, ± 40 ppm) were measured by an integrated sensor of Shandong Renke RS-CO2WS-N01-2. Total solar radiation (0–1800 W/m^2^, 1W/m^2^) was measured by a total radiation sensor (RS-RA-N01-AL). Soil parameters were obtained using a soil temperature and moisture sensor (Kong Saien KE-N01-TR-1), which recorded temperature and volumetric water content. The sensors were configured to synchronize the acquisition of data at 10-minute intervals via a centralized Modbus/RS-485 interface. structure of the greenhouse environmental data acquisition system is illustrated in Fig 2.

**Fig 3:**
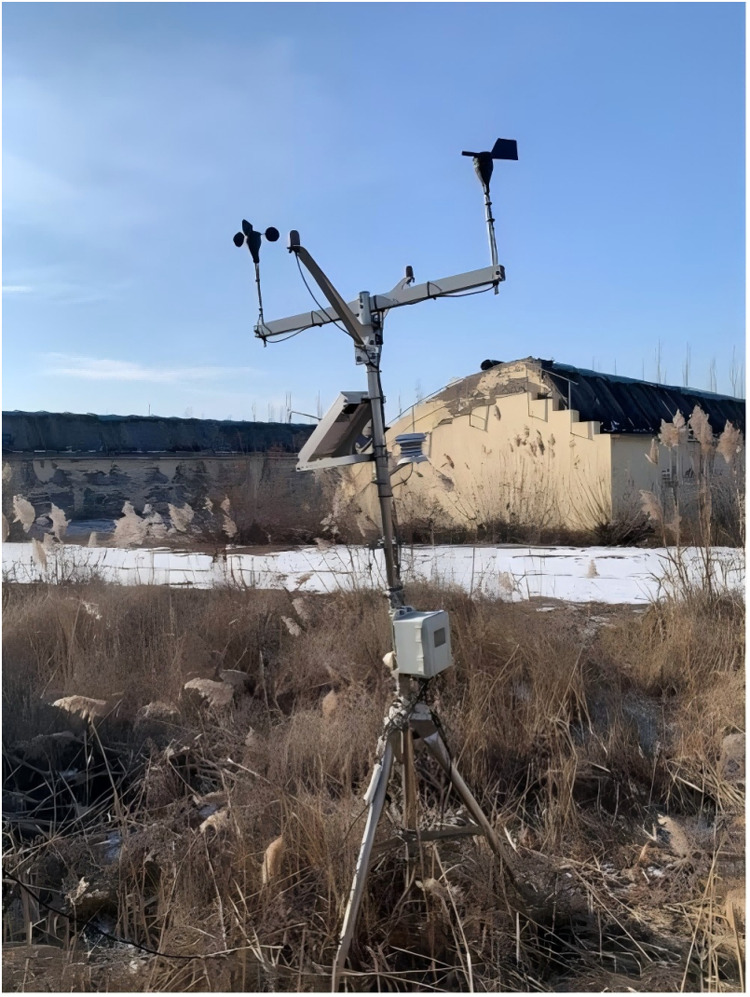
Weather station.

The data on indoor air temperature and relative humidity used in this paper were obtained from integrated sensors installed at the geometric center of the greenhouse (1.8 m above ground level, positioned at the intersection of east-west and north-south axes), and it was found that the data from this location could reflect the mean values of greenhouse temperature and humidity in a representative way. Soil temperature and moisture content data were obtained from sensors buried 5 cm below the soil surface at the same central location. The total solar radiation sensor was installed at the midpoint of the greenhouse’s east-west axis, 3 m from the southern film cover, and at a height of 1.3 m. This positioning minimizes structural shading effects while ensuring representative radiation data collection. The outdoor meteorological parameters are obtained from HOBO U30 NRC weather station installed in the open area outside the greenhouse (Fig 3).

### 2.2 Data sources

This study used a dataset from 22 December 2021 0:00–19 February 2022 14:30, for the training and testing of the model. The dataset contained the following variables: outdoor temperature (T_out_), outdoor relative humidity (RH_out_), outdoor wind speed (WS_out_), indoor temperature (T_in_), indoor relative humidity (RH_in_), indoor soil temperature (TS_in_) and soil water content (SWC_in_), indoor light intensity (PAR_in_). Data from 26 December 2021 8:10–17:40, 6 January 2022 19:40–7 January 2022 08:00 were missing due to power outages. Therefore, the total amount of data is equal to 8441 samples (as shown in S1 Data).

**Fig 4:**
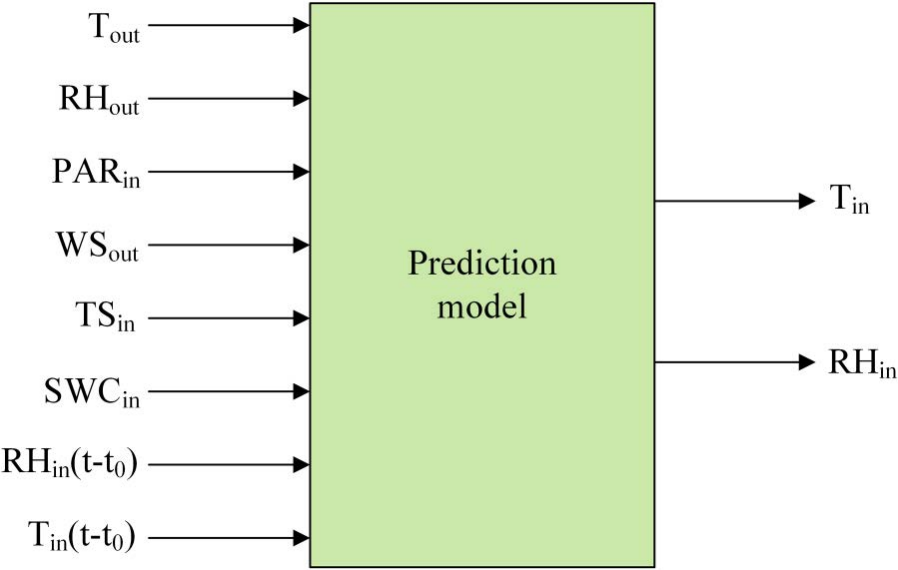
Configuration of the prediction model.

Temperature and humidity variations inside the greenhouse are influenced by both external climatic conditions and the greenhouse’s environmental factors. These variations exhibit a significant time lag. Consequently, the environmental data collected indoors and outdoors (T_out_, RH_out_, WS_out_, TS_in_, SWC_in_, PAR_in_), along with the historical indoor temperatures and relative humidity from the previous 10 minutes (RH_in_ (t-t_0_), T_in_ (t-t_0_), t_0_ = 10 min), were utilized as inputs for the prediction models. The outputs of the prediction model are the temperature and relative humidity inside the greenhouse after 30 minutes, as it balances the response lag of greenhouse environmental regulation with the cumulative effect of model errors – as the prediction time period lengthens, the system nonlinearity enhances leading to a decrease in prediction accuracy [12]. Configuration of the prediction model is illustrated in Fig 4.

### 2.3 MLP network

The core structure of the multilayer perceptron (MLP) is that of a classical feed-forward neural network, consisting of an input layer, a hidden layer, and an output layer [23]. The nonlinear mapping of multidimensional environmental parameters is realized by means of full connectivity. The input layer receives input variables including indoor and outdoor environmental factors, which are then transformed by the hidden layer through a non-linear process. The output layer then produces the predicted values of the target environmental factors. The MLP is capable of effectively capturing the complex interaction mechanisms among greenhouse environmental variables by adaptively adjusting the weight parameters through the back-propagation algorithm. While traditional training relies on gradient descent, advanced optimizers like the Levenberg-Marquardt (LM) algorithm can be employed. The LM algorithm enhances training by adaptively blending the Gauss-Newton method (utilizing second-order derivatives) and gradient descent through a damping factor, significantly accelerating convergence in nonlinear regression tasks. Consequently, the LM algorithm is employed in both the primary input variable selection and data division components of the experimental design. Furthermore, there are numerous alternative training algorithms, including Bayesian Regularization and Scaled Conjugate Gradient. A comprehensive list can be found in Table 1. In the following discussion, we propose a comparative analysis of different algorithms.

**Table 1:** Types of training algorithms.

Algorithm Name	Function
Levenberg-Marquardt	trainlm
Bayesian Regularization	trainbr
Scaled Conjugate Gradient	trainscg
Resilient Back-propagation	trainrp
Variable Learning Rate GD	traingdx
Adaptive Learning Rate GD	traingda
BFGS Quasi-Newton	trainbfg
Conjugate Gradient (Powell)	traincgb
Conjugate Gradient (Fletcher)	traincgf
Conjugate Gradient (Polak)	traincgp
One Step Secant	trainoss

### 2.4 RBF network

The Radial Basis Function (RBF) network is a three-layer feedforward neural network that utilizes radial basis functions as activation units in the hidden layer. Its architecture comprises an input layer for feature mapping, a nonlinear hidden layer with Gaussian kernels, and a linear output layer for regression or classification tasks [24]. The input layer size is equal to the number of model inputs, with an equivalent weight assigned to each input. Hidden layer neurons must be selected to determine the optimal number of neurons to ensure prediction effectiveness. The output layer size is equal to the network output, with two neurons in this study since the goal is to predict greenhouse temperature and relative humidity.

The network’s hidden layer utilizes Gaussian activation functions centered on prototype vectors to compute Euclidean distances from the input data. Gaussian kernel function that is calculated based on $\varphi_{i}$[20]:


$$\varphi_{i}(x)=e^{-\frac{{\left(x-c_{i}\right)}^{T}(x-c_{i})}{2\sigma_{i}^{2}}}$$ (1)

Where $\sigma_{i}$ is the width of RBF. The $c_{i}$ is the center of RBF.

The selection of the width parameter of function directly influences the local response range of the Gaussian kernel function, which is typically [25]:


$$\sigma =\frac{d_{\max}}{\sqrt{2h}}$$ (2)

Where d_max_ is the maximum Euclidean distance between all central vectors, and h is the number of hidden layer neurons.

The output layer performs linear combinations of these nonlinear transformations, it is weighted by hidden nodes, and the formula for calculation is as follows [20]:


$$y(x)=w_{i}+\sum_{i=1}^{h}w_{i}\varphi_{i}(x)$$ (3)

Where $w_{i}$ is the weight between the hidden and the output neurons, y(x) is the output.

### 2.5 Normalization

It is clear that environmental factors have neither the same units nor the same orders of magnitude. Thus, variables with higher values contribute more to the output error, with the result that the algorithm gives more weight to them and less to variables with a smaller range of values [26]. For this reason, data must be normalized before being presented to the network. Data normalization compresses the range of the data between 0 and 1. The normalization in this study was carried out via the following expression given by Eq. (4) [27].


$$X_{n}=\frac{X_{r}-X_{\min}}{X_{\max}-X_{\min}}$$ (4)

Where $X_{n}$ is the normalized value of $X_{r}$, $X_{r}$ is a real value, and $X_{\max}$d $X_{\min}$note the maximum and minimum values in the data, respectively.

### 2.6 Predictive model evaluation indicators

To verify the predictive performance of the BP neural network model for environmental factors in solar greenhouses, the determination coefficient (R^2^) and root mean square error (RMSE) were selected as model evaluation indices. The calculation equations of each evaluation index are as follows [28].

#### 1. Root mean square error (RMSE).

The root mean square error, also known as the standard error, reflects the degree of deviation between the true value and the predicted value.


$$\begin{array}{c} \text{RMSE}=\sqrt{\frac{\sum_{i=1}^{n}{\left(y_{i}-{\hat{y}}_{i}\right)}^{2}}{n}} \end{array}$$ (5)

#### 2. Determination coefficient (R^2^).

Where R^2^ indicates the closeness of the linear relationship between the predicted and measured values. The closer R^2^ is to 1, the better the model effect is. When the sum of squares of the prediction residuals is greater than the sum of squares of the variance of the true value, R^2^ becomes negative, indicating that the fitting effect is extremely poor and that the prediction effect is not as good as the direct mean value.


$$R^{2}=1-\frac{\sum_{i=1}^{n}{\left(y_{i}-{\hat{y}}_{i}\right)}^{2}}{\sum_{i=1}^{n}{\left(y_{i}-\overset{―}{y}\right)}^{2}}$$ (6)

In the above equations, n is the number of samples, $y_{i}$ is the measured value of the i-th sample, ${\hat{y}}_{i}$ is the predicted value of the i-th sample, and $\overset{―}{y}$ is the average of the measured values of the n samples.

### 2.7 Correlation analysis

There are nonlinear coupling relationships between different environmental factors and indoor temperature and indoor relative humidity. To enhance computational efficiency in modeling, spearman correlation analysis was used to initially identify the key input variables [29]. Table 2 shows the matrix of correlation coefficients between the two outputs and all input features. Correlation coefficients ranging from ±0.4 to ±1.0 generally reflect statistically significant associations, whereas values between −0.3 and +0.3 typically suggest weak or negligible relationships between variables [30], positive values indicate concordant variations, whereas negative values reflect inverse dependencies. Therefore, all input variables were selected except soil water content for indoor temperature and indoor relative humidity predictive model. Subsequently, sensitivity analyses will be performed on input variables other than SWC_in_ to further reduce the dimensionality of the prediction model and improve prediction efficiency.

**Table 2:** Correlation coefficient between all inputs and outputs.

	RH_in_(t-t_0_)	T_in_(t-t_0_)	T_out_	RH_out_	PAR_in_	WS_out_	TS_in_	SWC_in_
**RH**_**in**_**(t-t**_**0**_)	1.00							
**T**_**in**_**(t-t**_**0**_)	−0.92^**^	1.00						
**T**_**out**_	−0.56^**^	0.64^**^	1.00					
**RH**_**out**_	0.44^**^	−0.62^**^	−0.27^**^	1.00				
**PAR**_**in**_	−0.94^**^	0.85^**^	0.54^**^	−0.32^**^	1.00			
**WS**_**out**_	−0.54^**^	0.53^**^	0.46^**^	−0.38^**^	0.53^**^	1.00		
**TS**_**in**_	−0.25^**^	0.49^**^	0.23^**^	−0.75^**^	0.09^**^	0.11^**^	1.00	
**SWC**_**in**_	−0.02^*^	0.04^**^	−0.01	−0.11^**^	0.04^**^	0.06^**^	0.04^**^	1.00
**T**_**in**_	−0.93^**^	0.96^**^	0.61^**^	−0.53^**^	0.90^**^	0.54^**^	0.34^**^	0.03^**^
**RH**_**in**_	0.94^**^	−0.84^**^	−0.49^**^	0.33^**^	−0.94^**^	−0.51^**^	−0.10^**^	−0.00

** Correlation is significant at the 0.01 level;

* Correlation is significant at the 0.05 level.

## 3. Results and discussion

### 3.1 Input variable selection via sensitivity analysis

A systematic approach based on sensitivity analysis and multivariate combinatorial validation was used in this study to determine the optimal set of input variables for a greenhouse environment prediction model. First, the sensitivity index of each input variable to the output (temperature and humidity) was calculated by perturbation method to rank the importance of the variables: positive and negative perturbations were applied to each variable in the validation set, and the strength of the influence was evaluated by the amount of change in the mean square error of the feed-forward neural network output. Based on the ranking results of 10 independent experiments, the Kendall’s W (W = 0.98) concordance test was used to verify the stability of variable importance. Then, a progressive combination validation strategy was adopted to sequentially select the first k highly sensitive variables (k = 1, …, 7), build the MLP neural network, and obtain the optimal model through 20 times of random initialization training.

As illustrated in Table 3, the sequence of importance of the input variables is as follows: RH_in_(t-t_0_), PAR_in_, T_in_(t-t_0_), TS_in_, T_out_, RH_out_, WS_out_. Table 3 shows that the 4-variable combination (RH_in_(t-t_0_), PAR_in_, T_in_(t-t_0_), TS_in_) attains near-optimal predictive performance for both temperature and humidity. In terms of temperature prediction, the subset achieves an RMSE of 1.50°C (R^2^ = 0.96), thereby signifying an 81.5% error reduction in comparison to the single-variable baseline (RH_in_(t-t_0_) alone: RMSE = 2.43°C). In addition, the accuracy of the humidity prediction is demonstrated to improve to an RMSE of 3.95% (R^2^ = 0.95), which signifies a 57.1% error reduction from the baseline (9.20%). It can be seen that there is a substantial improvement in the prediction effect after the addition of TS_in_ to the input variables, indicating a strong interaction between soil temperature and air temperature and humidity in the greenhouse, Deng et al. [31] confirmed the existence of a complex coupling relationship between soil temperature and greenhouse air temperature and humidity in terms of dynamic energy exchange and feedback regulation by building an analytical model that integrates soil evaporation, solar radiation absorption, thermal radiation loss and convective heat transfer. Although the continued introduction of the variables (T_out_, RH_out_, WS_out_) led to a slight reduction in RMSE, the improvement in prediction was not significant. Notably, the five-variable model showed an increase in the RMSE of temperature prediction (1.50°C to 1.54°C), suggesting that redundant outdoor temperatures may lead to overfitting risk. This result suggests a reasonable balance between model simplicity and the preservation of physical relationships governing greenhouse environments. During subsequent parameter optimization phases for both MLP and RBF prediction models, these 4 variables were consistently employed as input parameters for greenhouse temperature and relative humidity prediction modeling.

**Table 3:** Prediction results for different combinations of input variables.

Variable	RMSE_T_in_ (°C)	R^2^_T_in_	RMSE_RH_in_ (%)	R^2^_RH_in_
**RH**_**in**_**(t-t**_**0**_)	2.43	0.89	9.20	0.70
**RH**_**in**_ **(t-t**_**0**_ **), PAR**_**in**_	1.77	0.94	6.04	0.87
**RH**_**in**_**(t-t**_**0**_**), PAR**_**in**_**,T**_**in**_**(t-t**_**0**_)	1.73	0.94	4.70	0.92
**RH**_**in**_ **(t-t**_**0**_ **), PAR**_**in**_ **,T**_**in**_ **(t-t**_**0**_ **), TS**_**in**_	1.50	0.96	3.95	0.95
**RH**_**in**_ **(t-t**_**0**_ **), PAR**_**in**_ **,T**_**in**_ **(t-t**_**0**_ **), TS**_**in**_ **, T**_**out**_	1.54	0.96	3.81	0.95
**RH**_**in**_ **(t-t**_**0**_ **), PAR**_**in**_ **,Tin(t-t**_**0**_ **), TS**_**in**_ **, T**_**out**_ **, RH**_**out**_	1.45	0.96	3.71	0.95
**All**	1.41	0.96	3.66	0.95

### 3.2 Data set partitioning

In order to rigorously evaluate the generalizability of MLP and RBF models while preventing overfitting, a comparative analysis of data partitioning strategies was conducted (80%−20%, 70%−30%, 60%−40% for training-testing splits). In order to ensure the stability of the results, K-fold cross-validation was used with K = 5, repeat 3 times. Prediction results presented in Table 4 (temperature) and Table 5 (relative humidity). It can be seen that when the training set is 80%, in the temperature prediction model, the RMSE of the MLP model is slightly higher on the test set, while the R^2^ of the RBF model is lower than that of the 70% training set; when the training set is 70% of the sample size, the MLP exhibits the best generalization ability for both temperature and humidity prediction (temperature test set RMSE = 1.59°C, humidity test set RMSE = 4.42%), whereas the RBF network has a higher R^2^ and lower RMSE in temperature prediction and the highest R^2^ in humidity prediction; When the proportion of the training set is reduced to 60%, the prediction performance of both the MLP and the RBF models is observed to decrease, indicating that an insufficient quantity of training data can result in inadequate learning by the model, which may consequently lead to under fitting. So, 70% sample size is finally selected as the best split.

**Table 4:** Temperature prediction results of MLP and RBF networks by different training sets.

Training size	Model	Train		Test	
		RMSE_T_in_ (°C)	R²_Tin	RMSE_T_in_ (°C)	R^2^_Tin
80%	MLP	1.42	0.967	1.70	0.944
	RBF	1.41	0.967	1.65	0.947
70%	MLP	1.53	0.960	1.59	0.958
	RBF	1.38	0.968	1.66	0.954
60%	MLP	1.38	0.966	1.73	0.954
	RBF	1.35	0.967	1.70	0.956

**Table 5:** Humidity prediction results of MLP and RBF networks by different training sets.

Training size	Model	Train		Test	
		RMSE_RH_in_ (%)	R²_RH_in_	RMSE_RH_in_ (%)	R²_RH_in_
80%	MLP	4.16	0.947	4.51	0.933
	RBF	3.99	0.951	4.50	0.933
70%	MLP	4.27	0.942	4.42	0.945
	RBF	3.89	0.952	4.63	0.939
60%	MLP	3.99	0.949	4.59	0.940
	RBF	3.80	0.953	4.67	0.938

### 3.3 MLP prediction model

This section explores the impact of training algorithm selection, the number of nodes in the hidden layer on model performance, in accordance with the predetermined optimal input variables and data partitioning strategy.

#### 3.3.1 Training algorithm selection.

**Fig 5:**
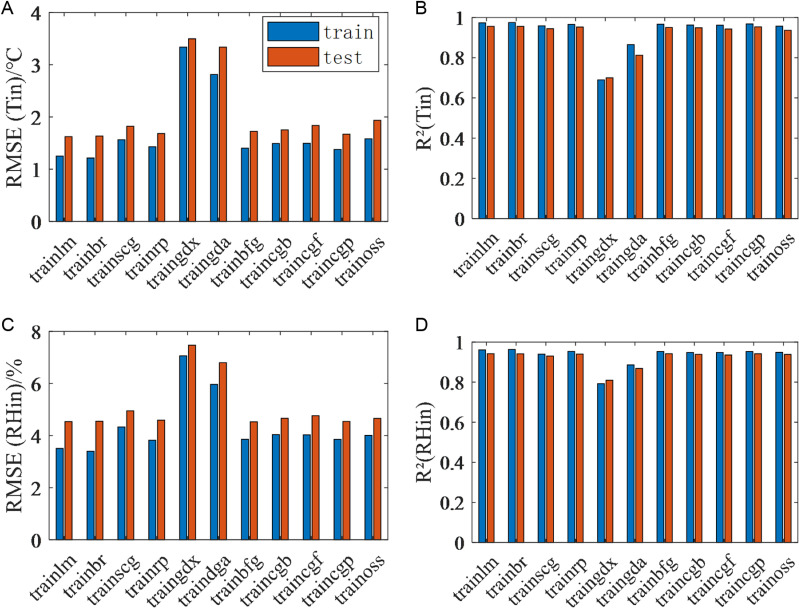
Mean RMSE and R^2^ for the training and test phases of MLP by different training algorithms for temperature and humidity prediction. (a) Mean RMSE of temperature prediction. (b) Mean R^2^ of temperature prediction. (c) Mean RMSE of humidity prediction. (d) Mean R^2^ of humidity prediction.

As illustrated in Fig 5, it is evident that the Levenberg-Marquardt and Bayesian regularization algorithms demonstrated optimal performance for both temperature and humidity. The RMSE and the R^2^ of the predicted temperature and humidity on the training and test sets are similar. This finding suggests that the system is capable of accurately capturing the primary characteristics of the input variables during the training phase and providing a high level of accuracy on the test set. This indicates excellent generalization ability and stability. The LM algorithm demonstrated the most superior performance in temperature prediction (RMSE = 1.624°C, R^2^ = 0.956) and humidity prediction (RMSE = 4.535%, R^2^ = 0.942). The Bayesian regularization algorithm ranked second, with an RMSE of 1.634°C, R^2^ of 0.955 in temperature prediction and an RMSE of 4.548%, R^2^ of 0.942 in humidity prediction. A comparative analysis of the generalization ability of the results reveals that the LM algorithm exhibits a slight advantage, thereby substantiating its selection as the optimal training algorithm for the Multi-Layer Perceptron (MLP). Conversely, the Variable Learning Rate Gradient Descent algorithm yields poor results.

#### 3.3.2 Number of hidden layer nodes selection.

In order to ascertain the optimal number of hidden layer nodes, a grid search method was employed to observe the change in performance of the predictive model when the number of hidden layer nodes was increased from 3 to 30. In order to enhance the reliability of the results, each node configuration was subjected to 10 independent repetitions of the experiment. The LM training algorithm was used, an early stopping mechanism was set (maximum number of failures is 6), and L2 regularization (λ= 0.005) was optimized to prevent over fitting.

**Fig 6:**
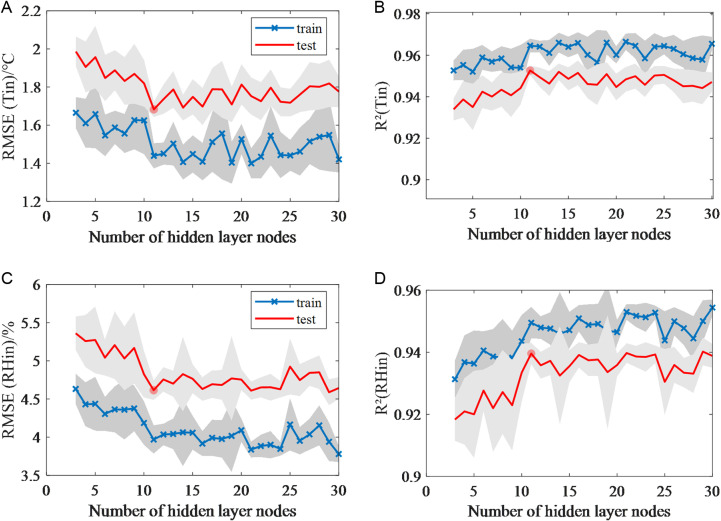
Mean RMSE and R^2^ for the training and test phases of different number of hidden layer nodes for temperature and humidity prediction. (a) RMSE for temperature prediction. (b) R^2^ for temperature prediction. (c) RMSE for humidity prediction. (d) R^2^ for humidity prediction.

The prediction results for temperature and relative humidity at different numbers of nodes are shown in Fig 6. When the number of hidden layer nodes is set at 11, the temperature prediction model achieves a test set RMSE of 1.68°C, with the narrowest confidence interval (as shown by the shaded band in Fig 6(a)). Concurrently, showing an R^2^ above 0.95, demonstrating robust performance. At 11 nodes, the humidity prediction model achieves an RMSE of 4.62%, with the narrower confidence interval bandwidth across tested configurations (Fig 6(c)), coupled with an R^2^ exceeding 0.93, indicating both high accuracy and stability.

As shown in table 6, the discrepancy in performance between the optimal node 11 and node 3 in temperature prediction has been substantiated through the implementation of a one-way analysis of variance (ANOVA) in conjunction with a Tukey’s HSD post-hoc test (significance level α = 0.05), thereby confirming a statistically significant outcome. The discrepancy in performance between the optimal node 11 and node 3 in temperature prediction was found to be statistically significant. Furthermore, no statistically significant difference was observed between the selected node 11 and the optimal node (node 29) in humidity prediction. Additionally, the system’s humidity prediction did not demonstrate a significant enhancement with an increase in the number of nodes. Thus, 11 nodes were selected for the MLP model, achieving optimal prediction accuracy for both temperature and humidity within a unified network.

**Table 6:** Temperature & Humidity ΔRMSE Differences (ANOVA-Tukey HSD).

Prediction	Node Pair	△RMSE	Significance(p-value)
Temperature	3-11	0.3035°C	0.0257*
	3-14	0.2940°C	0.0399*
Relative humidity	3-29	0.7723%	0.0380*

### 3.4 RBF prediction model

**Fig 7:**
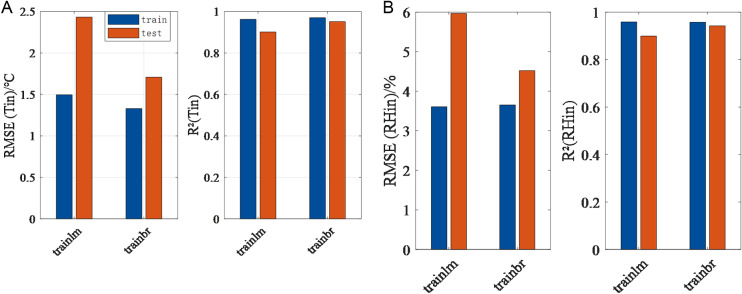
Mean RMSE and R^2^ for the training and test phases of RBF by different training algorithms for temperature and humidity prediction. (a) Temperature prediction. (b) Humidity prediction.

As with MLP, the selection of training algorithms has a direct impact on the accuracy of predictions. In the relevant literature, Taki et al. [32] used 13 different algorithms compared with regard to their ability to predict greenhouse temperatures, and it indicates that both the LM algorithm and the Bayesian algorithm have demonstrated satisfactory performance. The present study thus compares the impact of these two algorithms on the output results of the RBF prediction model, as illustrated in Fig 7. The Bayesian optimization algorithm has been shown to have a clear advantage in the RBF network, with higher R2 values and lower RMSE in temperature and relative humidity predictions. Consequently, the Bayesian algorithm is selected as the optimization algorithm for the RBF prediction model.

### 3.5 Optimal prediction model selection

The optimized MLP and RBF models were utilized to predict the temperature and relative humidity of the greenhouse over the following 30 minutes. The parameters of the MLP model are detailed in the preceding section, the RBF prediction model utilizes the Bayesian regularization optimization algorithm previously identified. The hidden layer RBF centers are initialized via K-means clustering, with the maximum number of RBF neurons is 300 based on empirical validation. During training, the hidden layer neuron count automatically adapts to the to the training sample size, while the spread parameter is dynamically adjusted via Eq. (2) to harmonize the expressive and generalization capabilities of the model. The maximum iterations capped at 500 (typically not reached, adjustable if required).

As summarized in Table 7, the RMSE and R^2^ values of both the MLP and RBF prediction models are presented for the training, validation, and test datasets. The standard deviation of RMSE for the RBF and MLP models on the validation set is ± 0.058 and ±0.074, respectively, demonstrating their robustness to data fluctuations. The high R^2^ value (0.958) and low RMSE can basically be maintained on the test set, proving that both models have good generalization ability. The RBF model demonstrates marginally superior performance, with its RMSE is 1.579°C, smaller than that of the MLP model. For humidity prediction, the MLP and RBF prediction models perform well on both the training and validation sets, with the R^2^ reaching about 0.96, and the RBF network outperforms the MLP network due to having a smaller RMSE and higher R^2^. However, the reversal of performance during the testing phase suggests that MLP may have superior generalization potential for humidity prediction. So, for humidity prediction, MLP model is preferred. The overall error of the temperature prediction model was significantly lower than that of the humidity prediction, which suggests that humidity varies in a large range (36%−98%) over the course of a day, and that its changing characteristics are more difficult to capture, for example, when encountering snowy weather or irrigation operations in the greenhouse, the changes are more different from sunny days and non-irrigation times. The MLP prediction model has a smaller RMSE and higher R^2^ on the test set. Jung et al. [12] employed an RNN-LSTM model for 30-minute-ahead prediction of temperature and humidity in a Venlo type multi-greenhouse, with an R^2^ of 0.96 for temperature, 0.80 for humidity.

**Table 7:** Prediction results of indoor temperature and humidity after 30 minutes.

Model & Prediction	Train		Validation		Test	
	RMSE	R²	RMSE	R²	RMSE	R²
MLP T_in_	1.317 ± 0.068	0.971 ± 0.003	1.338 ± 0.074	0.970 ± 0.003	1.584	0.958
RBF T_in_	1.205 ± 0.020	0.975 ± 0.001	1.203 ± 0.058	0.975 ± 0.002	1.579	0.958
MLP RH_in_	3.694 ± 0.148	0.956 ± 0.003	3.719 ± 0.247	0.956 ± 0.006	4.299	0.948
RBF RH_in_	3.325 ± 0.072	0.965 ± 0.002	3.445 ± 0.192	0.962 ± 0.004	4.501	0.943

**Fig 8:**
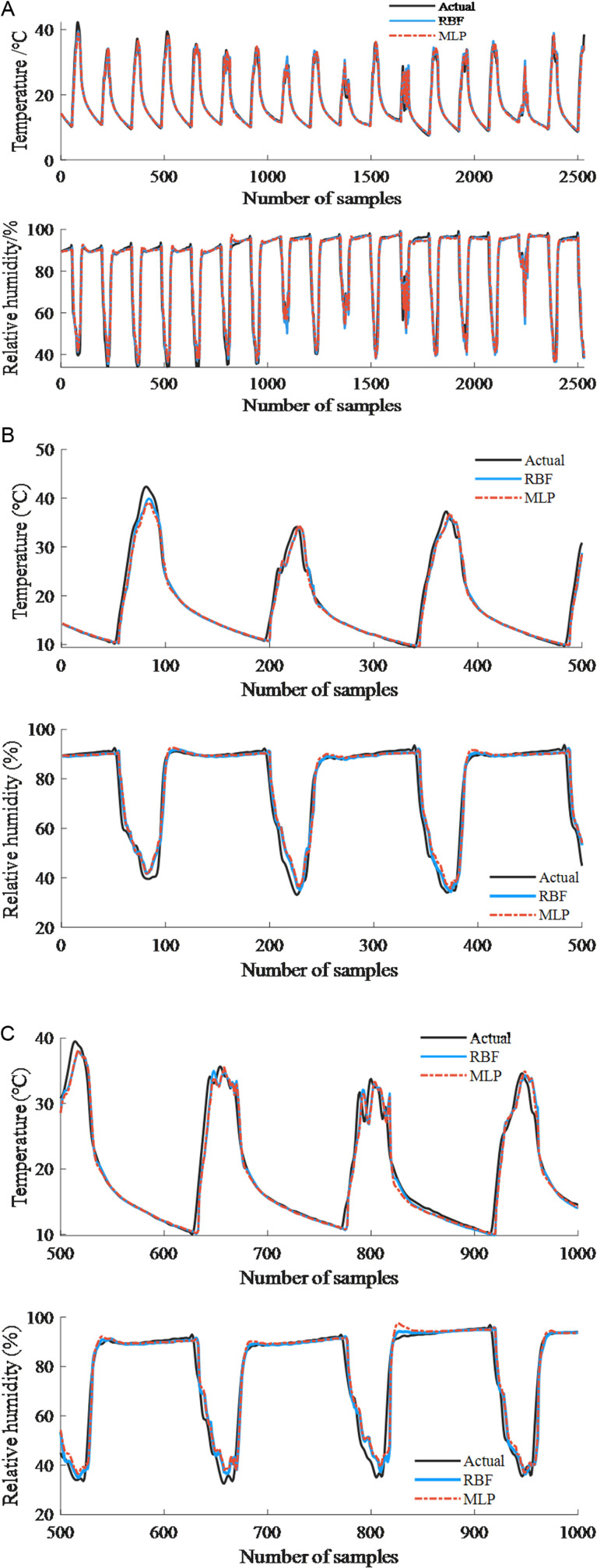
Comparison curve of predicted and actual values of MLP and RBF prediction models (30 minutes). (a) All testing sets. (b) 0-500 samples. (c) 500-1000 samples.

As shown in Fig 8, the comparison curves of predicted values and true values obtained using RBF and MLP prediction models are presented. Fig 8(a) illustrates the performance of prediction results across all test sets (2531). To clearly demonstrate the prediction scenarios under different data conditions, each segment of 500 samples is selected for local display, as shown in Figs 8(b) and 8(c).

From the comparison curves, it can be seen that the predicted values of the two models, MLP and RBF, are in good agreement with the real values as a whole. The time when the temperature and humidity prediction value have a large error with the measured value occurs during periods when the greenhouse temperature and relative humidity change drastically, for example, near the extreme value point, and the prediction accuracy of this location is affected by the change of greenhouse operation status. For example, when the insulating quilt was unfolded in the morning on a sunny day, the intensity of solar radiation continued to increase (peaking at noon), resulting in a sudden increase in greenhouse temperature and a sudden decrease in humidity [33]; superimposed on the secondary sudden change in temperature and humidity triggered by the opening of the vents at noon. The nonlinear coupling effect formed by such multiple dynamic perturbations poses a dual challenge to the model’s ability to capture rapidly changing processes.

**Fig 9:**
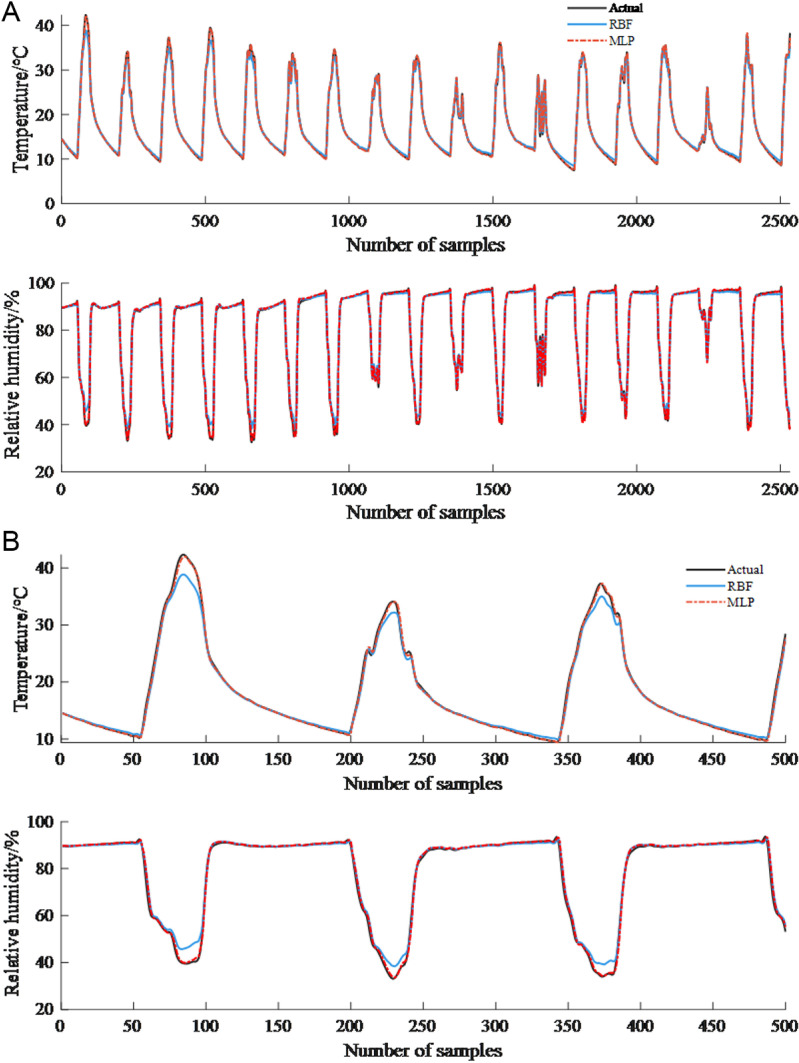
Comparison curve of predicted and actual values of MLP and RBF prediction models (current time). (a) All testing sets. (b) 0-500 samples.

In order to comprehensively assess the model performance, the prediction of temperature (T_in0_) and relative humidity (RH_in0_) in the greenhouse at the current moment was tested with the above model parameters, as shown in Table 8 for the performance of the prediction model on the training, validation and test sets, The consistently low RMSE and high R^2^ values of both models across all datasets validate their excellent stability and generalization capability in temperature prediction. It can be seen that the prediction of the MLP model is better than that of the RBF. Fig 9 shows the comparison between the prediction curves of MLP and RBF with actual values. Fig 9 (a) presents the curves for all prediction datasets, while Fig 9 (b) shows the local comparison curves for 0–500 sampling points. It can be seen that the prediction curve of the MLP model almost perfectly matches the actual values, whereas the RBF prediction model has larger deviations near the highest temperature and lowest humidity points. Therefore, in the case of real-time prediction of temperature and humidity in the greenhouse, the MLP prediction model has more obvious advantages in temperature and humidity prediction.

**Table 8:** Prediction results of current indoor temperature and humidity.

Model & Prediction	Train		Validation		Test	
	RMSE	R²	RMSE	R²	RMSE	R²
MLP T_in0_	0.511 ± 0.187	0.995 ± 0.004	0.520 ± 0.198	0.995 ± 0.004	0.439	0.997
RBF T_in0_	0.698 ± 0.167	0.991 ± 0.004	0.701 ± 0.172	0.991 ± 0.005	0.688	0.992
MLP RH_in0_	1.212 ± 0.163	0.995 ± 0.001	1.221 ± 0.143	0.995 ± 0.001	1.141	0.996
RBF RH_in0_	1.470 ± 0.202	0.993 ± 0.002	1.481 ± 0.236	0.993 ± 0.002	1.690	0.992

### 3.6 Discussion

This study systematically evaluated the ability of MLP and RBF neural network models in predicting greenhouse temperature and relative humidity for the next 30 minutes and verified the performance of the optimized models in predicting environmental factors at the current moment. By innovatively integrating correlation analysis, partial sensitivity analysis, and Kendall’s W coefficient of concordance, the input variables were optimized from 8 to 4 critical parameters. This variable reduction strategy not only mitigated potential errors from redundant sensors but also reduced hardware deployment costs.

Both prediction models were rigorously validated through 5-fold cross-validation and independent testing. For the prediction of temperature and humidity in the greenhouse at the current moment, both the MLP model and the RBF model showed good stability and generalization ability, and the MLP prediction model outperformed the RBF prediction model for temperature prediction with a smaller RMSE of 0.439°C and a higher R^2^ of 0.997 and for humidity prediction with an RMSE of 1.141% and R^2^ of 0.996. For the 30-minute advance forecast, the experimental results indicated that the RBF network demonstrated a superior capacity for temperature prediction,with an RMSE of 1.579°C and an R^2^ of 0.958. In contrast, the MLP model exhibited a higher degree of proficiency in humidity forecasting, with an RMSE of 4.299 and an R^2^ of 0.948. However, both prediction models exhibited significant error amplification during periods of rapid environmental fluctuation, such as those caused by ventilation activation or abrupt irradiance changes. This phenomenon was primarily attributed to their inability to effectively capture nonlinear coupling effects between multiple control actuators. While light intensity parameters did indirectly reflect Insulated quilts status, critical discrete variables such as ventilation on/off states were not incorporated in the current modeling.

Future research should prioritize integrating binary operational parameters of environmental actuators (ventilation/insulation) into input features and enhancing model mapping capabilities for abrupt state transitions. In addition, the overall effect of humidity prediction is somewhat worse than temperature prediction, and the prediction performance of relative humidity can be further improved in the future by optimizing the algorithm and input variables. This study lays a theoretical foundation for the intelligent regulation of greenhouse environmental factors in cold and arid regions, which is expected to enhance the efficiency of greenhouse management and yield optimization through precise environmental factor regulation. With the continuous development and expansion of facility agriculture, greenhouse precision environmental control system will have a broader application prospect.

## Supporting information

S1 Data Indoor and outdoor environmental data. (XLSX)
